# Health of Syrian unaccompanied asylum seeking adolescents (UASA) at first medical examination in Germany in comparison to UASA from other world regions

**DOI:** 10.1186/s12914-019-0192-8

**Published:** 2019-02-26

**Authors:** Annika Laukamp, Luise Prüfer-Krämer, Florian Fischer, Alexander Krämer

**Affiliations:** 1 School of Public Health, Bielefeld University, PO Box 100131, 33501 Bielefeld, Germany; 2 Travel Clinic, Furtwänglerstraße 9, 33604 Bielefeld, Germany

**Keywords:** Unaccompanied asylum seeking adolescents, Syria, Physical health, Mental health, Health behaviour, Refugee, Infections

## Abstract

**Background:**

The war in Syria has led to the biggest refugee crisis of our time. Unaccompanied asylum seeking adolescents (UASA) are a particularly vulnerable subgroup of refugees. This study aims to describe the weight status, health behaviour and disease spectrum of Syrian UASA in comparison to UASA from other world regions.

**Methods:**

The study was conducted as a prospective cross-sectional analysis of health metrics and diagnoses from 346 UASA (78% male; mean age 16 years) between 2011 and 2017. The data was collected in an outpatient clinic for internal and tropical medicine during a systematic medical examination after arrival. Descriptive and bivariate analyses stratified by gender and region/country of origin were performed.

**Results:**

The general health status of the UASA in most cases was good. Compared to other UASA Syrian UASA had the highest underweight prevalence (16.7%) (*p* = .013) and the second highest smoking prevalence (37.9%) (*p* < .001). Diseases at first medical examination mostly were infections and diseases of the digestive system, with significant differences between the regions/countries of origin (*p* < .001; *p* < .001, respectively). Syrian UASA had a lower prevalence of infections (28%) and a similar prevalence of mental and behavioural problems (10.3%) than the average of all regions/countries of origin (48.6%; 11%, respectively).

**Conclusion:**

Tailored screening for diseases as well as for health behaviour immediately after arrival in Germany is needed to reduce the individual burden of disease and to offer targeted preventive measures.

**Electronic supplementary material:**

The online version of this article (10.1186/s12914-019-0192-8) contains supplementary material, which is available to authorized users.

## Background

In recent years, persistent armed conflicts, and particularly the war in Syria, have caused an increasing number of refugees. UNHCR High Commissioner Filippo Grandi called the ongoing war in Syria the “biggest humanitarian and refugee crisis of our time” [1].

In 2016, 51% of the world’s refugees were children under the age of 18 years [2]. A particularly vulnerable subgroup of these are unaccompanied or separated children [3] because they are exposed to various health risks during the pre-flight, flight, and post-flight phases [4–6].

At the beginning of 2017, 47.990 UASA lived in Germany [7]. More than one quarter (28%) of the UASA that came to Germany in 2016 was from Syria [8] and the ongoing war in Syria may further increase the number of UASA applying for asylum in Germany during the next years. Furthermore, 98.4% of all Syrian UASA that applied for asylum in Germany in 2016 got a permit to stay [8] with the perspective of an integration into the German society to a greater extent compared to other asylum seeking groups.

An important task within Germany is the integration of these UASA. Following Ager and Strang (2008), successful integration is linked to the achievement of and access to several key domains [9]. One important key domain is health. For that reason, the aim of this study is to identify the weight status, health behavior and disease spectrum among UASA with a special focus on Syrian UASA. Most of the previous studies on health of UASA have concentrated on infectious diseases at the time of arrival [10–12], on mental diseases [13], or had small sample sizes [14].

This study is the first comparing the weight status, health behavior and the disease spectrum after arrival of Syrian UASA with UASA coming from other world regions. Knowing which UASA have risky behaviours and knowing their disease spectrum is important to adopt the German health care system and to sensitise the different professional groups working with UASA.

## Methods

### Study design

The study was conducted as a prospective cross-sectional analysis of health metrics and diagnoses. Convenience sampling was used for a non-representative subset of UASA in Germany. The UASA that were accommodated in so-called “Clearinghouses” in Bielefeld received a systematic first medical examination after arrival in one particular outpatient clinic for internal and tropical medicine in Bielefeld between September 2011 and January 2017. We included all these 346 UASA in our analysis. Within this outpatient clinic the first medical examination followed a systematic protocol. The examinations were performed by a medical doctor with long-term experience in treating migrants being board certified in Internal Medicine, Tropical Medicine, and Infectious Diseases in Germany.

### Systematic medical examination

The systematic first medical included the following components: (a) medical history, (b) complete physical examination, (c) laboratory diagnostics of blood and stool, and (d) tuberculosis screening. During the first medical examination, a caregiver/interpreter was on site and verbal consent was obtained from the UASA and/or caregiver.

- The medical history included information about age, country of origin, region of origin (according to the sub-regions defined by the United Nations [15], which are North Africa, sub-Saharan Africa, West Asia, South Asia, and “other regions” [comprising South Europe, South-East Asia, and Central Asia]), duration of flight, flight route, length of stay in Germany, current symptoms, symptoms indicating mental problems (e.g. sleeping disorders, lack of appetite, weight loss), alcohol, nicotine (cigarettes per day) and drug consumption, and physical activity.
- A complete physical examination, including the patient’s weight, height and body-mass-index (BMI), neurological examination, and blood pressure. BMI was categorised according to BMI reference curves for children and adolescents (percentile curves for the BMI) using the recommended categories:
  underweight: BMI under the reference percentile < 10;
  normal weight: BMI between the reference percentile 10–90;
  overweight and obesity BMI over the reference percentile > 90 [16, 17].
- Stool examination was performed for Samonellae, Shigellae, Campylobacter, Aeromonas, and Vibrios. Detection of worm eggs, larvae, and protozoa were performed by MIFC and EIAs for *Giardia lamblia* and *Entamoeba histolytica* antigen. Blood-cell count plus differential blood count and routine blood chemistry tests were carried out. Serology included the detection of HBsAg, anti-HBc, anti-HBS, HCV antibodies, H.pylori antibodies and for UASA originating or travelling through endemic regions, antibody-screening for Schistosoma was added.
- According to the German Infection Protection Act §36 article 4 tuberculosis screening was done by X-ray for UASA who are at least 16 years old [18]. For UASA younger than 16 years or for pregnant UASA Gamma-Interferon-Test was performed [19].

An overview about all aspects that were captured as part of the medical history and the physical examination is given within the screening document [see Additional file 1]. All diagnoses that were made within the first two quarters of medical care were included in the analysis, because in some cases follow-up examinations were necessary to get a reliable diagnosis. These diagnoses were classified based on ICD-10-GM 2017.

### Statistical analysis

Differences between genders and regions/countries of origin in relation to health behaviour and the classified diagnoses at first medical examination, are presented using significance tests (Kruskal-Wallis test by ranks, Wilcoxon rank-sum test, Pearson’s chi-squared test and Fisher’s exact test) at a significance level of 5%. Stata SE version 12 was used for analysis.

## Results

### Sociodemographic characteristics

In total, 346 UASA (78% male) underwent the medical protocol. The mean age was 16 years for males (SD ±1.06) and females (SD ± 1.26) (*p* = .730). The UASA within our study originated from 32 countries. The most common countries were Syria (19.8%), Iraq (17.2%) and Afghanistan (11.7%). Therefore, the most common regions of origin were West Asia (38.2%), South Asia (22.7%), and sub-Saharan Africa (20.4%) (Table 1).

**Table 1:** Countries of origin of the UASA

Regions of origin	Countries of origin
West Asia (*n* = 131)	Syria (*n* = 68); Iraq (*n* = 59); Georgia (*n* = 4)
South Asia (*n* = 78)	Afghanistan (*n* = 40); Bangladesh (*n* = 17); Iran (*n* = 9); Sri Lanka (*n* = 5); India (*n* = 4); Pakistan (*n* = 2); Nepal (*n* = 1)
sub-Saharan Africa (*n* = 70)	Guinea (*n* = 25); Somalia (*n* = 18); Eritrea (*n* = 12); Senegal (n = 4); Ethiopia (*n* = 3); Gambia (*n* = 2); Chad (*n* = 1); Ghana (*n* = 1); Mali (*n* = 1); Nigeria (*n* = 1); Rwanda (*n* = 1); Sierra Leone (*n* = 1)
North Africa (*n* = 47)	Morocco (*n* = 27); Algeria (*n* = 15); Libya (*n* = 4); Tunisia (*n* = 1)
East Asia (*n* = 2)	Mongolia (*n* = 2)
South-east Asia (*n* = 1)	Myanmar (*n* = 1)
Central Asia (*n* = 5)	Tajikistan (*n* = 4); Uzbekistan (*n* = 1)
South Europe (*n* = 9)	Albania (*n* = 6); Serbia (*n* = 3)

Significant differences were identified between male and female UASA regarding their region/country of origin (*p* < .001). Most of the 74 female UASA came from sub-Saharan Africa (39%; *n* = 29), West Asia (19%; *n* = 14, without Syrian UASAs), Syria (16%; *n* = 12), and South Asia (16%; *n* = 12). Most males came from South Asia (25%; *n* = 66), Syria (21%; *n* = 56), and West Asia (18%; *n* = 49, without Syrian UASAs).

### Weight status and health behaviour

For 333 UASA BMI could be assessed (260 males; 73 females). Mean BMI was 18.85 kg/m^2^ (range: 11.88–31.61 kg/m^2^; SD ± 3.02). Overall, 84.4% showed normal weight, 8.4% were underweight, and 7.2% were overweight or obese (Table 2). Significant differences could be identified between the regions/countries of origin (*p* = .013) but not between males and females regarding BMI (*p* = .077). Compared with the other regions of origin, Syrian UASA had the highest underweight prevalence (16.7%).

**Table 2:** Weight status and health (risk) behaviours

	Total	Gender			Region/country of origin						
		F	M	p	SSA	other WA	SYR	SA	NA	O^a^	p
Underweight	8.4%	9.6%	8.1%	.077	13.6%	11.3%	16.7%	0%	2.2%	0%	.013
	28/333	7/73	21/260		9/66	7/62	11/66	0/75	1/45	0/17	
Normal weight	84.4%	76.7%	86.5%		80.3%	79%	74.2%	94.7%	93.3%	94.1%	
	281/333	56/73	225/260		53/66	49/62	49/66	71/75	42/45	16/17	
Overweight	7.2%	13.7%	5.4%		6.1%	9.7%	9.1%	5.3%	4.4%	5.9%	
	24/333	10/73	14/260		4/66	6/62	6/66	4/75	2/45	1/17	
Consume of alcohol	6.2%	1.4%	13.3%	.056	3.28%	12.1%	4.8%	0%	11.9%	11.8%	.012
	19/306	1/71	18/135		2/61	7/58	3/63	0/64	5/42	2/17	
Smoking	23.4%	5.3%	28.7%	<.001	3%	8.5%	37.9%	6.9%	71.7%	35.3%	<.001
	77/329	4/75	73/254		2/67	5/59	25/66	5/72	33/46	6/17	
Drug use	2.6%	1.4%	2.9%	.478	1.6%	0%	0%	1.5%	13.6%	0%	<.001
	8/310	1/71	7/239		1/61	0/58	0/63	1/66	6/44	0/17	
Physical activity	60.8%	30.9%	69.3%	<.001	50.8%	48.3%	65%	69.2%	75.6%	52.9%	.020
	188/309	9.6%	8.1%		32/63	28/58	39/60	45/65	34/45	9/17	

^a^Comprises UASA from South Europe, South-East Asia and Central Asia

SSA = sub-Saharan Africa; other WA = West Asia without Syrian UASA; SYR = Syrian UASA; SA = South Asia; NA = North Africa; O=Other Regions

6.2% of the UASA (135 males; 71 females) consumed alcohol, with significant differences between regions/countries of origin (*p* = .012). The prevalence of alcohol consumption was highest among West Asians without Syrian UASA (12.1%). Only 4.8% of the Syrian UASA consumed alcohol. Alcohol consumption was more prevalent in males than in females (13.3% vs. 1.4%; *p* = .056).

Smoking status was assessed for 329 UASA (254 males; 75 females). Overall, 23.4% of UASA smoked. Smoking was more prevalent in males than in females (28.7% vs. 5.3%; *p* < .001). The differences between regions/countries of origin was significant (*p* < .001). 71.7% of North African UASA smoked. In comparison to North African UASA the smoking prevalence of the Syrian UASA was lower (37.9%).

Overall, only 2.6% of the 310 UASA (239 males; 71 females) consumed drugs. Drug consumption was only found among UASA from North Africa (13.6%), sub-Saharan Africa (1.6%) and South Asia (1.5%) (*p* < .001) and did not differ between males and females (*p* = .478).

60.8% of the 309 UASA (241 male and 68 female) were physically active. Male UASA were more often physically active than females (69.3% vs. 30.9%; p < .001). Differences between the regions/countries of origin were significant (*p* = .020) with highest prevalence among North African UASA (75.6%). Syrian UASA were slightly more often physically active (65%) than the average of all regions/countries of origin (60.8%).

### Diagnoses

During the 346 first medical examinations, 860 diagnoses were made (males: 2.3 diagnoses on average (SD ± 1.89) vs. females: 3 diagnoses on average (SD ± 2.13) (*p* = .012). Differences between the regions/countries of origin were also significant (*p* < .001). On average Syrian UASA had 2 diagnoses (SD ± 1.37).

The classification of the diagnoses showed that 48.6% of the UASA had one or more infections. The prevalence of UASA with one or more infections differed significantly between UASA from different regions/countries of origin (*p* < .001). Infections were more than twice as frequent in sub-Saharan African UASA (74.3%) as in UASA from West Asia (without Syrian UASA) (27%) or Syria (28%).

31.8% had one or more diseases of the digestive system. Diseases of the digestive system differed significantly between the regions/countries of origin (p < .001). Overall, Syrian UASA had the lowest prevalence of diseases of the digestive system (17.7%). The most common disease within this category was gastritis (26.9%).

27.5% of the UASA suffered from iron deficiency or other nutritional diseases. Significant differences between males and females could be identified within this category (*p* = .001), with a much higher prevalence in female UASA (43.4%) than in males (23%). Differences between the regions/countries of origin and iron deficiency (*p* = .592) and iron deficiency anaemia (*p* = .848) were not significant. Within the category iron deficiency and other nutritional diseases iron deficiency (11.9%) and iron deficiency anaemia (11.3%) were the most prevalent diseases.

Mental and behavioural problems were detected in 11% of the UASA. Mental and behavioural problems did not differ significantly between the regions/countries of origin (*p* = .811) or male and female UASA (*p* = .330). The diagnoses within this category were mostly posttraumatic stress disorder (PTSD) (4.6%) and depression (2.9%) (Table 3).

**Table 3:** Prevalences (%) in UASA with one or more diseases within the most common diagnosis categories

ICD-10 Chapters	Total	Gender			Region/country of origin						
		F	M	p	SSA	other WA	SYR	SA	NA	O	p
Infections ^a^	48.6	55.3	46.7	.185	74.3	27	28	60.3	49	47.1	<.001
Diseases of the digestive system ^b^	31.8	42.1	28.9	.029	57.1	19.1	17.7	33.3	29.8	35.3	<.001
Iron deficiency & nutritional diseases ^c^	27.5	43.4	23	<.001	32.9	33.3	23.5	25.6	21.3	29.4	.597
Diseases of the respiratory system ^d^	14.7	17.1	14.1	.510	14.3	17.5	16.2	16.7	10.6	5.9	.792
Diseases of the skin ^e^	12.4	9.2	10	.838	18.6	12.7	7.4	15.4	8.5	5.9	.353
Injury, poisoning & external causes ^f^	11.3	9.2	11.9	.520	10	11.1	13.2	12.8	10.6	0	.777
Mental and behavioural problems ^g^	11	7.9	11.9	.330	11.4	9.5	10.3	9	14.9	17.7	.811

Total (*n* = 345); F=Female UASA (*n* = 76); M = Male UASA (*n* = 270); SSA = sub-Saharan Africa (*n* = 70); other WA = West Asia without Syrian UASA (*n* = 63); SYR = Syrian (*n* = 68); SA = South Asia (*n* = 78); NA = North Africa (*n* = 47); O=Other Regions (*n* = 17)

^a^Infections (ICD 10-I): see Table 4

^b^Diseases of the digestive system (ICD 10-XI) comprising mostly gastritis (26.9%) & chronic hepatitis (3.2%)

^c^Iron deficiency & nutritional diseases (ICD 10-IV & ICD 10-III) comprising mostly iron deficiency (11.9%) &

iron deficiency anaemia (11.3%)

^d^Diseases of the respiratory system (ICD 10-X) comprising mostly virus infection of upper respiratory tract

(5.8%) & tonsillitis (2.9%)

^e^Diseases of the skin (ICD 10-XII) comprising mostly acne (3.8%) & dermatitis (not specified) (3.2%)

^f^Injury, poisoning & external causes (ICD 10-XIX) comprising mostly contusion (3.2%) & fractures (1.5%)

^g^Mental and behavioural problems (ICD 10-V) comprising mostly PTSD (4.6%) & depression (2.9%)

The most prevalent infections were *H. pylori* infections (34.5%), helminth infections (8.7%) and Giardiasis (5.3%) (Table 4). Significant differences were found for *H. pylori* infections in UASA from different regions/countries (*p* = .001). UASA from Syria had the lowest prevalence (19%) of *H. pylori* infections and sub-Saharan UASA had the highest (53.9%).

**Table 4:** Prevalences (%) of the most common infections

Infections	Total	Gender			Region/country of origin						
		F	M	p	SSA	other WA	SYR	SA	NA	O	p
*H. pylori*	34.5%	40.3%	33.3%	.292	53.9%	24.5%	19%	34.4%	37.5%	46.7%	=.001
	104/298	27/67	77/231		35/65	13/53	11/58	22/64	15/40	7/15	
one or more Helminths ^a^	8.7%	10.5%	8.2%/	.515	27.1% ^b^	4.8%	0%	6.4%	6.4%	0%	<.001
	30/346	8/76	22/270		19/70	3/63	0/68	5/78	3/47	0/17	
Giardiasis	5.3%	7.1%	4.7%	.432	6.1%	1.7%	5%	9.2%	2.8%	0%	.526
	16/302	5/70	11/232		4/66	1/59	3/60	6/65	1/36	0/13	
Scabies	4.2%	5.3%	4.4%	.764	10%	1.6%	2.9%	2.6%	6.4%	5.9%	.197
	16/346	4/76	12/270		7/70	1/63	2/68	2/78	3/47	1/17	
Hepatitis B	4.2%	2.8%	4.6%	.518	13.4%	0%	0%	6.4%	0%	0%	<.001
	14/335	2/71	12/264		9/67	0/63	0/64	5/78	0/46	0/15	
Mycosis	3.5%	0%	4.4%	.076	1.4%	0%	2.9%	7.7%	6.4%	0%	.114
	12/346	0/76	12/270		1/70	0/63	2/68	6/78	3/47	0/17	
Amoebiasis	3.3%	5.6%	2.6%	.255	7.7%	0%	1.7%	2.9%	5.6%	0%	.204
	10/304	4/72	6/232		5/65	0/58	1/60	2/68	2/36	0/14	

*SSA* sub-Saharan Africa, other *WA* West Asia without Syrian UASA, *SYR* Syrian, *SA* South Asia, *NA* North Africa, *O*=Other Regions

^a^Comprising: Schistosoma, Hymenolepis nana, Ancylostomatidae, Oxyuridae, Trichuris trichiura, Strongyloides

^b^High prevalence due to Schistosoma infections that were only tested in UASA from sub-Saharan Africa

Further Infections: Gastroenteritis (1.5%), latent tuberculosis (1.5%), verrucas (1.2%), Aeromonas caviae (0.9%), herpes (0.9%), Campylobacter (0.3%), Epstein-Barr virus (0.3%), varicella zoster (0.3%), Haemophilus influenzae (0.3%), cryptosporidia (0.3%), lice (0.3%), post-polio syndrome (0.3%), Tularemia (0.3%)

Significant differences could also be detected for helminthic infections with respect to the regions/countries of origin (*p* < .001). Helminthic infections were common in UASA from sub-Saharan Africa (27.1%). The high proportion of helminthic infections among sub-Saharan African UASA was due to Schistosomiasis. 65 of the sub-Saharan Africa UASA were tested for Schistosomiasis and 24.6% of them were infected. Among Syrian UASA no helminthic infection was detected.

The prevalence of Hepatitis B virus infection overall was 4.2%, with significant differences between the regions/countries of origin (p < .001). Hepatitis B virus infections were only detected among UASA from sub-Saharan Africa (13.4%) and South Asia (6.4%).

Five cases of latent tuberculosis infection were diagnosed among the UASA (two from South Asia, two from North Africa, and one from sub-Saharan Africa).

## Discussion

The results indicated that, overall, UASA had a good health status, showed some risky health behaviours but have had a complex burden of diseases at time of first medical examination shortly after arrival in Germany. Syrian UASA had a lower disease burden than the average of all UASA in some of the reported disease categories.

On average, 84.6% of UASA had a normal weight status, 8.4% were underweight, and only 7.2% were overweight or obese. Comparing these with the weight of adolescents from Germany (age 14–17 years), it becomes clear that in German adolescents the prevalence of overweight and obesity is much higher (17.1%), and the prevalence of underweight lower (6.7%). Syrian UASA had the highest prevalence of underweight (16.7%) within this study and this prevalence is more than twice as high compared with German adolescents (age 14–17 years) (6.7%) [20]. These differences between Syrian UASA and German adolescents cannot be explained by a lower background prevalence of overweight and/or a higher prevalence of underweight in Syria [21]. It probably can be explained by the long duration of the flight conditions, which causes exhaustion and poor access to food and due to adaptation and mental problems in Germany.

Smoking was the most common (23.4%) health risk behaviour among UASA, with male UASA having a significantly higher smoking prevalence than females (28.7% vs. 5.3%). In comparison, German adolescents showed a higher prevalence, but smaller gender differences (39% male and 31% female adolescents) [22]. A study on the smoking prevalence of 16-year-old high-school students in Syria from 2000 showed that 15.9% of the males and 6.6% of the females were current smokers [23]. The male Syrian UASA in our study (37.9%) showed a much higher smoking prevalence than their male peers in Syria (15.9%). This discrepancy may indicate that smoking is kind of a coping strategy used by male Syrian UASA to deal with their war and flight experience. Furthermore, it may indicate that the Syrian UASA which were able to finance the escape from Syria, came from more privileged families and thus are able to finance smoking.

Overall, only 6.2% of the UASA reported alcohol consumption. A comparable prevalence (7%) was found in Syrian adults in a study from Fouad et al. in 2006 [24]. In the KiGGs-Study from Germany, it was pointed out that 27.9% of adolescents (age 14–17 years) showed risky alcohol consumption [25]. Possible reasons for the low prevalence among UASA could be religious rules. Although religion was not formally assessed in our study, it can be assumed that a vast majority of Asians are Muslims.

North African UASA had the highest prevalence of smoking (71.7%) and drug consumption (13.6%) in this study, whereas Syrian UASA had only a high smoking prevalence (37.9%). These UASA in particular should be focussed for preventive measures and interventions.

60.8% of UASA were physically active (69.3% of males and 30.9% of females), with a higher prevalence among North Africans (75.6%). A recent study from Musaiger and Kalam (2014) among Syrian adolescents in Damascus also described a significantly higher physical activity among males than females (88.2% vs. 64.2%) [26]. The lower amount of physical activity among Syrian UASA in this study (65%) compared to Syrian adolescents in Syria may be explained by a phase of recovery from exhaustion and/or new orientation after the flight experience.

Overall, the UASA within our study showed several health-risk behaviours that could be tackled with preventive measures. These preventive measures should especially focus on smoking, drug consumption and physical inactivity. An essential criteria for the effectiveness of preventive measures is the accessibility of the target group [27]. An advantage of UASA is that most of them are easy to reach because they are accommodated in so-called “Clearinghouses” in Germany. Therefore, there is the option of implementation of preventive measures as setting-based low threshold approaches within the “Clearinghouse”. The aim of setting-based approaches is to create a supportive environment that enables good health [28], for example a smoking free Clearinghouse or regular physical activity within the Clearinghouse. Due to the diversity of the Clearinghouses and the UASA such approaches should be adapted to the local circumstances. Over the years the involved Clearinghouses have already implemented offers of physical activity and psychological counselling for their UASA.

The diagnoses that were made during the first medical examination were mostly infections. These findings are in accordance with other studies in UASA and adult asylum seekers in Germany [10–12, 29]. These infections represent mainly the epidemiology of their regions/countries of origin. The high burden of helminthic diseases among UASA from sub-Saharan Africa (27.1%) is largely due to Schistosoma infections, due to the high endemic rates in this region. A high prevalence of intestinal parasites was described by Mockenhaupt et al. (2016) in UASA from Syria. 22% of the 488 UASA had at least one intestinal parasitic infection [12]. In our study Syrian UASA showed a relative low burden of infections in comparison to UASA from other regions of origin. However, 28% of the Syrian UASA in our study had at least one infection. *H. pylori* infection (19%) and Giardiasis (5%) were the most common infections among Syrian UASA within our study. A slightly higher prevalence of Giardia duodenalis was reported by Theuring et al. (2016). They screened 500 Syrian UASA between 2014 and 2015 in Berlin and 7.2% of them were infected with Giardia duodenalis [10]. The differences between the studies may be caused by different screening techniques or frequencies, e.g. examination of three stool specimen compared to only one in our study.

The overall high prevalence of infections may be explained by poor hygiene, high background prevalences, poor access to health care before flight and poor hygienic conditions during and after flight, e.g. in overcrowded camps [4]. Iron deficiency, as a marker for poor nutritional status, which was found in our study population, can attribute to the susceptibility for infectious diseases [30]. Differences between male and female UASA were found regarding iron deficiency and other nutritional diseases. Also Jablonka et al. (2018) reported a significant higher prevalence of moderate anaemia among refugee women than among men (8.2% vs. 1.2%: *p* < .001) [31]. Iron deficiency and iron deficiency anaemia especially occur in women, because women have a higher demand for iron that can be explained through blood loss during menstruation. The circumstances before and during flight may also lead to nutritional deficiencies [30].

The mental and behavioural problems in our study were mostly PTSD (4.6%) and depression (2.9%). In a systematic review the prevalences of mental problems among UASA in Germany were higher and varied between 9 and 44%, depending on the methods and instruments used. The review showed that mental problems did not improve over time, which may indicate a transition into chronicity [13]. To prevent this development, it is necessary to identify mental diseases as early as possible. Therefore, standardised and binding guidelines regarding the content of a medical screening for UASA after arrival should include a psychological screening.

According to the German Infection Protection Act §36, article 4, all asylum seeker that are admitted to “collective accommodation” centres must provide a medical certificate indicating the absence of infectious pulmonary tuberculosis. For all refugees with a completed age of 15, this must be provided via X-ray examination [18]. A limitation of this legal regulation is that only pulmonary tuberculosis can be detected by X-ray examination. For adolescents and children under the age of 16, chest X-ray based screening is less sensitive and specific for pulmonary tuberculosis compared to adults. Furthermore, the use of ionising radiation should be kept as low as possible in this age group. Gamma-Interferon-Test or tuberculosis skin test is recommended for asylum seeker under the age of 16 [19]. We used Gamma-Interferon-Test for UASA younger than 16 years or for pregnant UASA, because it is easier to handle than the tuberculosis skin test. In contrast to the national recommendations the European Centre for Disease Prevention and Control (ECDC) recommends to offer screening for active tuberculosis and for latent tuberculosis but only for migrant populations from high tuberculosis incidence countries [32]. As a limitation, it must be mentioned that mental diagnoses in our study were not all made by a specialist which may question the validity. The information regarding health behaviour was self-reported and may be influenced by social desirability. Furthermore, informal interpreters were used. This could have influenced the responses of UASA, especially regarding sensitive questions referring to mental health problems, alcohol or drug use. Another limitation is that there were no specific definitions of the health behaviours. The UASA were asked, whether they consume drugs and alcohol and whether they were doing physical activity on a regular basis. Also, the study population is possibly not representative for Germany as a whole, because only UASA from Bielefeld were included. Therefore, generalisability of the results is limited. Considering that the medical examinations were done in an outpatient clinic with a focus on internal and travel medicine, only severe mental health problems were identified at the first visit. Despite these limitations, the results of this study indicate that UASA from Syria show a lower disease burden compared to UASA from other regions of origin regarding infections and diseases of the digestive system but showed the same burden of mental diseases as other UASA.

## Conclusion

Target-group-specific screening for infectious diseases, diseases of the digestive system, iron deficiency and mental problems directly after arrival is highly recommended to reduce the individual burden of disease and prevent the transition into chronicity. Screening for infectious diseases should not blindly be applied but depend on the a-priori probability of a defined infection in the respective (sub-)population which depends upon the prevalence of disease in the country of origin as well as in those countries where refugees had stayed during their flight. The Public health guidance on screening and vaccination for infectious diseases in newly arrived migrants within the EU/EEA from the ECDC already summarizes the evidence and gives recommendations regarding different infectious disease screenings in newly arrived migrants [32]. From an ethical perspective a mandate for treatment (albeit possibly expensive) is required in case of an infection. In Germany, currently a confusing patchwork of different screening strategies exists which differ between states and even counties and from a public health perspective there is no reasoned explanation for these differences [33]. Within their nationwide analysis of state policies regarding health examination of asylum seekers in Germany, Wahedi et al. (2017) pointed out, that the indicators for certain screening strategies were often unclear [33]. Therefore, a tailored screening model should be developed that can be standardised and used for appropriate and evidence-based individual care of UASA and other asylum seekers. This philosophy should similarly apply to other disease entities.

We encourage longitudinal studies focusing on the development of diseases and health behaviour of UASA over time to identify UASA at high risk of poor health. This enables the above mentioned development of target-group-specific preventive measures that could help to sensitise UASA in their behaviour as well as help adapt the German health care system and therefore enable a better health status, and successful integration of this important refugee subgroup.

## Additional file

Additional file 1: Screening_document_medical_examination_UASA.pdf. (DOCX 188 kb)

## Abbreviations

ECDC: European Centre for Disease Prevention and Control
PTSD: Posttraumatic stress disorder
UASA: Unaccompanied asylum seeking adolescents

## References

[1] United Nations High Commissioner for Refugees (UNHCR). Syria emergency. 2017. http://www.unhcr.org/syria-emergency.html. Accessed 25 Apr 2018.

[2] United Nations High Commissioner for Refugees (UNHCR). Global Trends. Forced displacement in 2016. Geneva: United Nations High Commissioner for Refugees; 2017.

[3] Echavez C, Bagaporo JLL, Pilongo LH, Azadmanesh S (2014). Why do children undertake the unaccompanied journey?.

[4] Zimmerman C, Kiss L, Hossain M. Migration and health: a framework for 21st century policy-making. PLoS Med. 2011. 10.1371/journal.pmed.1001034. 10.1371/journal.pmed.1001034 PMC3101201 21629681

[5] Hebebrand J, Anagnostopoulos D, Eliez S, Linse H, Pejovic-Milovancevic M (2016). Klasen H. A first assessment of the needs of young refugees arriving in Europe: what mental health professionals need to know. Eur Child Adolesc Psychiatry.

[6] Huemer J, Karnik N, Steiner H (2009). Unaccompanied refugee children. Lancet.

[7] Huber A, Lechner C (2017). Die Situation unbegleiteter minderjähriger Geflüchteter in Deutschland.

[8] Bundesministerium für Familie, Senioren, Frauen und Jugend. Daten zu unbegleiteten minderjährigen Flüchtlingen. 03.02.2017. https://kleineanfragen.de/bundestag/18/11080-daten-zu-unbegleiteten-minderjaehrigen-fluechtlingen.pdf. Accessed 17 Dec 2018.

[9] Ager A, Strang A (2008). Understanding integration: a conceptual framework. J Refug Stud.

[10] Heudorf U, Karathana M, Krackhardt B, Huber M, Raupp P, Zinn C. Surveillance for parasites in unaccompanied minor refugees migrating to Germany in 2015. GMS Hyg Infect Control. 2016. 10.3205/dgkh000265 10.3205/dgkh000265 PMC4773540 26958459

[11] Theuring S, Friedrich-Jänicke B, Pörtner K, Trebesch I, Durst A, Dieckmann S (2016). Screening for infectious diseases among unaccompanied minor refugees in Berlin, 2014-2015. Eur J Epidemiol.

[12] Mockenhaupt FP, Barbre KA, Jensenius M, Larsen CS, Barnett ED, Stauffer W, et al. Profile of illness in Syrian refugees: a GeoSentinel analysis, 2013 to 2015. Euro Surveill. 2016. 10.2807/1560-7917.ES.2016.21.10.30160. 10.2807/1560-7917.ES.2016.21.10.30160 PMC12631605 26987893

[13] Witt A, Rassenhofer M, Fegert JM, Plener PL (2015). Hilfebedarf und Hilfsangebote in der Versorgung von unbegleiteten minderjährigen Flüchtlingen. Kindheit und Entwicklung.

[14] Marquardt L, Krämer A, Fischer F, Prüfer-Krämer L (2016). Health status and disease burden of unaccompanied asylum-seeking adolescents in Bielefeld, Germany: cross-sectional pilot study. Tropical Med Int Health.

[15] United Nations Statistics Division (UNSD). Standard Country or Area Codes for Statistical Use (M49) - Global Inventory of Statistical Standards. 2011. https://unstats.un.org/unsd/iiss/Standard-Country-or-Area-Codes-for-Statistical-Use-M49.ashx. Accessed 6 Apr 2018.

[16] Arbeitsgemeinschaft Adipositas im Kinder- und Jugendalter (AGA). Definition der Adipositas [Definition of obesity]. http://www.aga.adipositas-gesellschaft.de/index.php?id=210. Accessed 28 May 2017.

[17] Arbeitsgemeinschaft Adipositas im Kinder- und Jugendalter (AGA). Was ist der Body-Mass-Index? [What is the body mass index?]. http://aga.adipositas-gesellschaft.de/mybmi4kids/. Accessed 24 May 2017.

[18] Bundesministerium der Justiz und für Verbraucherschutz. Gesetz zur Verhütung und Bekämpfung von Infektionskrankheiten beim Menschen: IfSG; 25.07.2017.

[19] Ritz N, Brinkmann F, Feiterna-Sperling C, Hauer B, Haas W. Tuberkulosescreening bei asylsuchenden Kindern und Jugendlichen ‹ 15 Jahren in Deutschland. Monatsschr Kinderheilkd 2015;163:1287–1292.

[20] Kurth B-M, Schaffrath Rosario A (2007). The prevalence of overweight and obese children and adolescents living in Germany. Results of the German health interview and examination survey for children and adolescents (KiGGS). Bundesgesundheitsblatt Gesundheitsforschung Gesundheitsschutz.

[21] Nasreddine L, Mehio-Sibai A, Mrayati M, Adra N, Hwalla N (2010). Adolescent obesity in Syria: prevalence and associated factors. Child Care Health Dev.

[22] Kuntz B, Lampert T (2016). Smoking and passive smoke exposure among adolescents in Germany. Dtsch Arztebl Int.

[23] Maziak W, Mzayek F (2000). Characterization of the smoking habit among high school students in Syria. Eur J Epidemiol.

[24] Fouad M, Rastam S, Ward K, Maziak W (2006). Prevalence of obesity and its associated factors in Aleppo, Syria. Prev Control.

[25] Lampert T, Kuntz B (2014). Tabak- und Alkoholkonsum bei 11- bis 17-jahrigen Jugendlichen: Ergebnisse der KiGGS-Studie - Erste Folgebefragung (KiGGS Welle 1). Bundesgesundheitsblatt Gesundheitsforschung Gesundheitsschutz.

[26] Musaiger A, Kalam F (2014). Dietary habits and lifestyle among adolescents in Damascus, Syria. Ann Agric Environ Med.

[27] Walter U, Salman R, Krauth C, Machleidt W (2007). Migranten gezielt erreichen: Zugangswege zur Optimierung der Inanspruchnahme präventiver Massnahmen. Psychiatr Prax.

[28] Dooris M (2006). Healthy settings: challenges to generating evidence of effectiveness. Health Promot Int.

[29] Bozorgmehr K, Mohsenpour A, Saure D, Stock C, Loerbroks A, Joos S, Schneider C (2016). Systematische Übersicht und “Mapping” empirischer Studien des Gesundheitszustands und der medizinischen Versorgung von Flüchtlingen und Asylsuchenden in Deutschland (1990-2014). Bundesgesundheitsblatt Gesundheitsforschung Gesundheitsschutz.

[30] World Health Organization (WHO). Iron Deficiency Anaemia: Assessment, Prevention and Control. A guide for programme managers. Geneva: World Health Organization; 2001.

[31] Jablonka A, Wetzke M, Sogkas G, Dopfer C, Schmidt RE, Behrens GMN, Happle C. Prevalence and types of Anemia in a large refugee cohort in Western Europe in 2015. J Immigr Minor Health. 2018. 10.1007/s10903-018-0725-6. 10.1007/s10903-018-0725-6 29582203

[32] European Centre for Disease Prevention and Control (ECDC). Public health guidance on screening and vaccination for infectious diseases in newly arrived migrants within the EU/EEA. Stockholm: European Centre for Disease Prevention and Control; 2018.

[33] Wahedi K, Nöst S, Bozorgmehr K (2017). Die Gesundheitsuntersuchung von Asylsuchenden: Eine bundesweite Analyse der Regelungen in Deutschland : § 62 Asylverfahrensgesetz. Bundesgesundheitsblatt Gesundheitsforschung Gesundheitsschutz..

[34] Piasecki J, Waligora M, Dranseika V (2017). What do ethical guidelines for epidemiology say about an ethics review? A qualitative systematic review. Sci Eng Ethics.

