# Supplementary material for: Health of Syrian unaccompanied asylum seeking adolescents (UASA) at first medical examination in Germany in comparison to UASA from other world regions
Source: BMC Int Health Hum Rights. 2019 Feb 26;19:5. doi: 10.1186/s12914-019-0192-8 (PMC6390530; doi:10.1186/s12914-019-0192-8)
Supplement: Supplementary file 1 — Screening_document_medical_examination_UASA.pdf. (DOCX 188 kb) [file 12914_2019_192_MOESM1_ESM.docx]

| **Dr. med. Luise Prüfer-Krämer**  **Internal and Tropical medicine** | 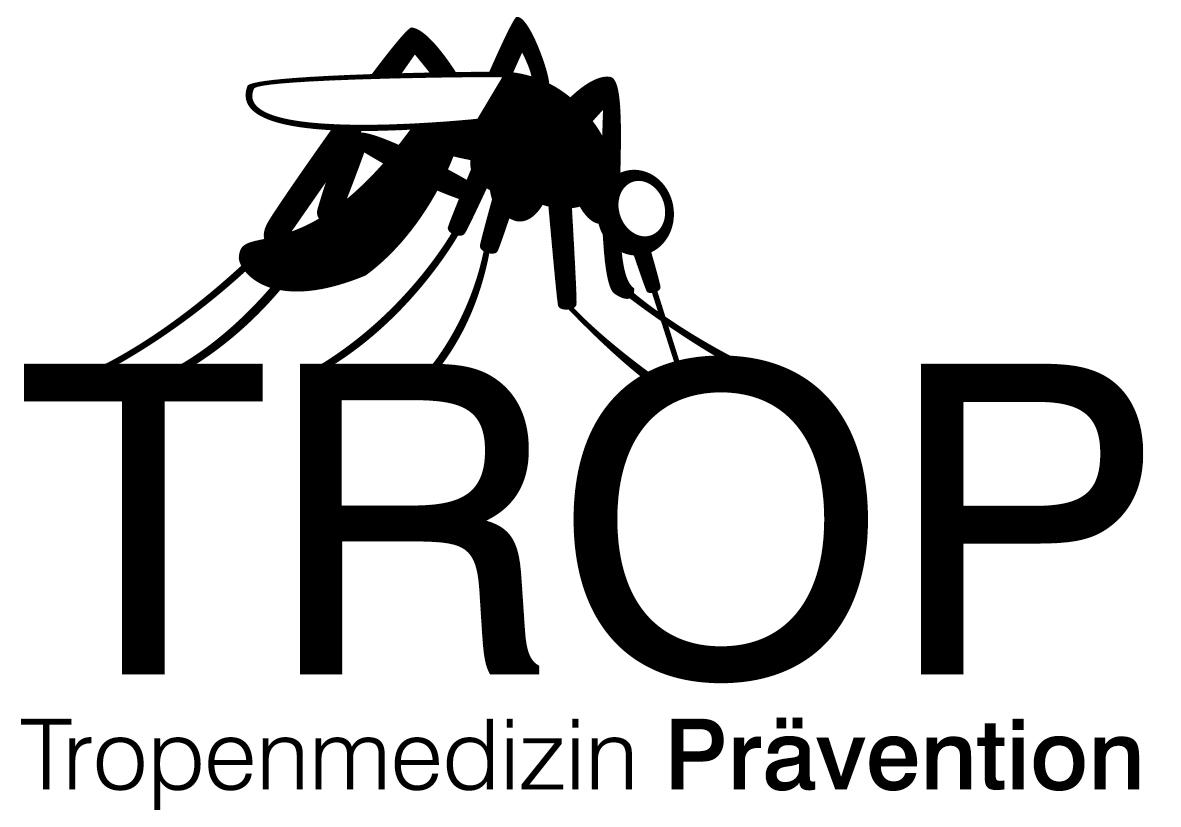 | **Furtwängler Str. 9**  **33604 Bielefeld** |
| --- | --- | --- |

|  | Date of the examination: |
| --- | --- |
|  | Last name: |
|  | First name:  Date of birth: |

**First medical examination of UASA**

**country of origin ______________________________________________**

**date of entry to Germany _______________________________**

flight route _______________________________

duration of flight _____________________________

medical history:

current health problems:

previous diseases:

previous stay(s) in a hospital:

previous treatment of tuberculosis: yes/no

allergies:

eating pattern:

sleep:

nightmares:

headache:

sadness:

physical activity: yes/no/if yes which?

last menstrual period (for girls):

nicotine: yes/no if yes: how many cigarettes per day:

medication: yes/no if yes: what kind of medicine:

alcohol: yes/no if yes: quantity:

drugs: yes/no if yes: what kind of drugs:

familiy (where?):

**Physical examination**

age:____years; gender: male/female; weight:____kg; height:____m; BMI:_____ kg/m²

| **general condition:** | thorax |
| --- | --- |
| **nutritional status:** | thorax form: |
|  | lung: |
| **skin:** |  |
|  |  |
| **mucous membranes:** |  |
|  | abdomen |
| head | tenderness: |
| nerve exits | liver: |
| eyes: pupils: | spleen: |
| light reflex: | kidneys: |
|  | **spine:** |
| ears: |  |
| nose: | extremities |
| mouth: dental status: | joints: |
| pharynx: | varices: |
| tonsils: | oedema: |
| tongue: |  |
|  |  |
| **neck** | nervous system |
| thyroid gland: | reflexes |
|  |  |
| cardiovascular |  |
| heart murmur: |  |
| rhythm: | **lymph nodes:** |
| pulse:____ /min. | **psyche:** |
| RR: ______mmHg |  |
|  |  |
| **annotations/other examinations:** | |
